# Advances in Imaging Specific Mediators of Inflammatory Bowel Disease

**DOI:** 10.3390/ijms19092471

**Published:** 2018-08-21

**Authors:** Nicole Dmochowska, Hannah R. Wardill, Patrick A. Hughes

**Affiliations:** Centre for Nutrition and GI Diseases, Adelaide Medical School, University of Adelaide and South Australian Health and Medical Research Institute, Adelaide 5000, Australia; nicole.dmochowska@adelaide.edu.au (N.D.); hannah.wardill@adelaide.edu.au (H.R.W.)

**Keywords:** Inflammatory Bowel Disease (IBD), colitis, PET, SPECT, microbiota, cytokine, chemokine, inflammation

## Abstract

Inflammatory bowel disease (IBD) is characterized by chronic remitting and relapsing inflammation of the lower gastrointestinal tract. The etiology underlying IBD remains unknown, but it is thought to involve a hypersensitive immune response to environmental antigens, including the microbiota. Diagnosis and monitoring of IBD is heavily reliant on endoscopy, which is invasive and does not provide information regarding specific mediators. This review describes recent developments in imaging of IBD with a focus on positron emission tomography (PET) and single-photon emission computed tomography (SPECT) of inflammatory mediators, and how these developments may be applied to the microbiota.

## 1. Introduction

Inflammatory bowel disease (IBD) is an inflammatory disorder of the gastrointestinal (GI) tract that impacts substantially on quality of life. The causes of inflammation in IBD remain unknown, but are thought to involve a hypersensitive immune response to the intestinal microbiota. IBD is commonly associated with dysbiosis, but it remains unknown whether dysbiosis is a cause or consequence of inflammation. Diagnosing and monitoring IBD is heavily reliant on endoscopy, which is an invasive technique that requires bowel preparation and typically also anesthesia. Although generally well tolerated, endoscopy noticeably impacts on patient quality of life and does not provide any direct information regarding the role that specific mediators contribute toward inflammation. Therefore, new technologies are required that are sensitive enough to grade disease severity, are non- or only minimally invasive, and can be optimized for the detection of specific mediators. Recent developments in positron emission tomography (PET), particularly with the use of antibody-conjugated tracers, have demonstrated success in cancers, but have only recently been adapted to IBD. This review summarizes the current advances of these technologies in IBD, how they have been used to detect specific mediators of inflammation, and their potential to image microbiota.

## 2. Inflammatory Bowel Disease

IBD is a collection of debilitating idiopathic diseases characterized by chronic inflammation of the lower gastrointestinal tract that have a remitting and relapsing disease course. The cause(s) of IBD remain unknown; however, they are thought to involve aberrant immune responses to environmental stimuli in people with a complex genetic predisposition. The global prevalence of IBD is estimated to be 0.3% and is widely regarded as increasing [1]. The relatively recent increase in the incidence of IBD is perplexing and highlights the likely importance of yet-to-be-determined environmental factors in its etiology. IBD is typically diagnosed in adolescence and early adulthood; however, it can also occur in the very young pediatric population, and approximately 25% of pediatric cases present prior to reaching 10 years of age [2,3]. It is currently unclear whether the mechanisms underlying IBD differ between the very young pediatric and late adolescent populations. However, it is probable that yet-to-be-characterized genetic factors feature more prominently in the very young, as the length of time of exposure to environmental factors is unlikely to be long enough to sensitize the developing immune system. IBD is a chronic disease in pediatric and adult populations; however, the pediatric population is particularly vulnerable to the consequences of IBD, including impacts on growth and development, bone health, and psychosocial function and development [2]. 

There are two major subtypes of IBD: Crohn’s disease (CD) and ulcerative colitis (UC), which can typically be distinguished by pathological and histological differences. Inflammation in UC is typically restricted to the mucosal layer of the colon and progresses in a contiguous manner. CD differs in that it is characterized by transmural skip lesions that can occur anywhere in the GI tract but are typically located in the ileum with and without involvement of the colon. The initiating factors and inflammatory aspects are likely to differ between CD and UC given the distinctions between their pathology, but are only poorly understood relative to what is known regarding how inflammation is perpetuated. This increased understanding has led to the relatively recent development of biologic drugs targeting specific immune mechanisms, most prominently the cytokines TNF-α, interleukin (IL)-12 and -23, and integrins involved in the migration of immune cells into the GI tract, including α_4_β_7_ [4]. These treatments and other nonspecific immune suppressants can be beneficial in IBD; however, treatment gaps remain due to intolerance, incomplete efficacy, and side effects of current therapies, leading to a disease course characterized by variable periods of remission and relapse. Prognostic indicators of relapse in IBD are only poor, and therefore patients are required to undertake constant surveillance, typically via endoscopy. Severe persistent or relapsing symptoms also lead to fibrosis and stricture in >30% of CD and approximately 5% of UC patients [5]. Intestinal fibrosis and stricture occurs as a result of excessive deposition of extracellular matrix proteins that accumulate following tissue remodeling and lead to serious complications, including obstructions [6]. No drugs are currently clinically available to treat fibrosis and stricture, and therefore these cases often lead to surgery, which can be curative for UC (withstanding complications), but is not curative for CD [7]. This high rate of surgery highlights the ongoing need for a deeper understanding of the mechanisms involved in initiating and perpetuating inflammation in IBD. 

### Imaging IBD: Current Approaches

Treatments for IBD depend on clinical severity, which is typically assessed by endoscopy. The gastrointestinal mucosa is directly imaged by endoscopy for assessment of disease stage and monitoring, and when forceps are attached, which allows for the collection of biopsy material for analysis of pathology and mechanistic studies. Endoscopy can differentiate between UC and CD in the majority of cases [8]. However, endoscopic approaches have limitations, as the quality of results is operator-dependent and it may not be sensitive enough for assessment of clinical severity in some cases. Furthermore, endoscopic imaging is restricted to the superficial mucosal layers of the intestine and therefore provides no information regarding inflammatory damage to the deeper layers of the intestinal wall, the degree of muscle thickness, or the diameter of the lumen. Finally, a major limitation of endoscopy is the difficulty in reaching the small intestine, due to the distance needed to be covered and the complexities of intestinal anatomy with its multiple loops and folds. These limitations are particularly relevant to the pediatric population, who are sensitive to perforation of the intestinal wall and risks associated with sedation [9]. 

A number of novel imaging technologies are emerging that complement or may even replace endoscopy as the gold standard for clinical diagnosis, as recently reviewed in detail [10,11]. Chromoendography involves the application of dyes onto the mucosa, improving endoscopic characterization of lesions and neoplasia, and has the potential to correlate with histological damage, although the latter remains controversial [12]. Confocal laser endomicroscopy and endocytoscopy combine high-resolution microscopic imaging with endoscopy, and again, are primarily used to detect colonic dysplasia and neoplasia [13]. Wireless capsule technologies may circumvent the limitations that endoscopy has in accessing difficult-to-reach areas of the small intestine. While these capsules provide high-resolution imaging, they have similar limitations to standard endoscopy regarding assessment of inflammatory damage to the deeper layers of the colon wall and may also require surgery for removal when stricture occurs [14,15]. 

Less invasive imaging technologies include barium X-rays, ultrasound, magnetic resonance imaging (MRI), computed tomography (CT), PET, and single photon emission computed tomography (SPECT). Barium X-rays provide excellent visualization of the bowel and can reveal thickening of the bowel wall, but are not recommended in patients with severe inflammation due to the risk of complications such as toxic megacolon, and while they have been traditionally used to assess stricture location and severity, this is largely being replaced by CT and MRI [2]. Ultrasound, MRI, CT, PET, and SPECT are minimally invasive relative to endoscopy and offer the additional benefits of visualizing planes through the colon wall and extraintestinal manifestations. Bowel ultrasound has some attractions as a noninvasive diagnostic tool for IBD, including relative inexpensiveness, high accuracy in CD, and ease of use. It has recently been used to reveal ulcer location and severity in adult CD, and ultrasound and MRI are currently the preferred tools for assessment of pediatric perianal abscesses and fistulas [16,17,18]. However, ultrasound is highly operator-dependent and its accuracy is sensitive to anatomical location, as it is reduced for detecting ulcers in the jejunum and rectum [17,18]. MRI is currently the imaging technique of choice for follow-up of CD patients due to its lack of ionizing radiation. Notably, MRI can differentiate between normal and pathological sections of the bowel by analysis of changes in bowel wall thickness [19]. Recent developments with MRI and CT include using them to assess fibrosis and stricture. Gadolinium-contrasted MRI has recently been shown to reliably predict severe fibrosis in CD and, interestingly, also revealed that areas of fibrosis often coexist with inflammation within the same intestinal segment [20]. A recent comprehensive review of the use of PET/SPECT for IBD diagnosis, prognosis, and follow-up indicated that 2-deoxy-2-[^18^F]fluoro-d-glucose (^18^F-FDG) coupled with CT has the highest accuracy for detecting inflammation in IBD [11]. ^18^F-FDG is a radiolabeled glucose analogue that detects tissue glucose metabolism and has been the tracer of choice for PET studies for decades; however, radiolabeled leukocytes are also typically used to assess GI inflammation. Indeed, SPECT of technetium-99m (^99m^Tc)-leukocytes was shown nearly two decades ago to accurately assess the extent and severity of disease for people with severe UC, correlating with endoscopic and histological findings [21]. However, limitations with radiolabeled leukocytes exist, including a relatively extensive protocol required for working up the cells, risks of cross-contamination, and importantly, the nonspecific nature of the tracer. Only limited information exists regarding the use of PET, SPECT, or CT for pediatric IBD, although the combination of ^18^F-FDG PET/CT has been suggested to be the most reliable due to its improved anatomical mapping [22]. Importantly, ^18^F-FDG dosing and imaging protocols are yet to be standardized for PET of pediatric patients, highlighting the need for more studies in this area. Nonetheless, the potential benefits of PET/CT in the pediatric IBD population were highlighted in the first published case, where scans in a 12-year-old child revealed diffuse heterologous uptake of ^18^F-FDG in the gut wall, particularly in the stomach [23]. CT confirmed that the uptake was focal, and endoscopy revealed ulceration of the gastric wall, with an ensuing diagnosis of gastric and intestinal CD. It is important to note that while these technological advances are welcome for IBD management, none of them currently target specific mediators. This highlights the need for the development of new imaging technologies that are highly sensitive, quantitative, and able to provide longitudinal data in real time, but are also only minimally invasive. 

## 3. Imaging Inflammatory Mechanisms in IBD: Which Targets to Choose?

### 3.1. Immune Targets

Inflammation is central to IBD; however, much remains to be understood regarding the causes of inflammation in humans. It is currently thought that inflammation in IBD is driven by a loss of immune tolerance to autologous proteins in the colon wall and foreign antigens in the lumen, including the microbiota. This initiates a cascade of cytokines and chemokines which cause an influx of immune cells, as comprehensively reviewed elsewhere [24,25]. Immune cells are attracted to sites of inflammation by chemokines and also by upregulated expression of “gut-homing” integrins on the cell surface, including α_4_β_7_ [26]. Subsequent inflammatory lesions and damage to the epithelial wall and deeper layers of intestinal tissue result in the production of damage-related mediators during active disease, which progresses toward mediators related to tissue-remodeling and wound healing as disease improves toward remission, but also toward mediators associated with fibrosis after repeated or extended periods of inflammation. The immunological basis for the differences in pathophysiology of UC and CD are not clear, and while it is suggested that CD is driven by a T_H_1/T_H_17-type immune response and UC is mediated by a T_H_2-type immune response (↑ IL-5, IL-13) that is atypical as IL-4 is not involved, much of the evidence for this generalization results from animal models. Furthermore, while there is evidence supporting a genetic predisposition, large-cohort IBD human studies indicate this is clearly complicated and contains loci that coexist in both CD and UC, but also other loci that are not shared [27,28]. Environmental factors are thought to contribute to IBD; however, evidence for or against these are heavily biased toward animal studies and are yet to be definitively determined in humans. The best evidence supporting an aggravating role for specific immune mediators in IBD is the success of several biologic medications targeting the cytokines TNF-α and IL-12/23 and the gut-homing integrin α_4_β_7_. This success is tempered by the lack of efficacy of therapies directed against IL-17, IFN-γ, and IL-13, amongst others [29]. However, as failure of biologics in the clinic can be related more to toxicity and side effects than to a lack of involvement of their target, tracers directed to these cytokines may still prove to be valuable for imaging. With this in mind, new immune modulators are currently undergoing clinical trials, including those targeting specific cytokines such as IL-6, inhibitors of intracellular pathways including Janus kinases (JAK), and mediators related to tissue remodeling and fibrosis, such as matrix metalloproteases (MMP) [29]. 

Recent advances in two-photon laser scanning microscopy have enabled in vivo imaging of deep visceral tissues and immune cells in particular, indicative of the potential for this technique to provide preclinical evidence prior to relatively expensive immuno-PET studies. Two-photon laser scanning microscopy was instrumental in identifying goblet cell-associated passage (GAPs) as the major mechanism for delivering luminal antigens to dendritic cells in the small intestine in health [30]. Interestingly, GAPs only appear in the colon following the disruption of microbe-sensing pathways, highlighting a potential role in IBD [31]. We have recently applied two-photon laser scanning microscopy to investigate immune cell migration in the trinitro benzene sulphonic acid (TNBS) model of colitis, demonstrating in vivo that not only are immune cells in very close apposition to nerves within the colon wall, but also that myeloid cell infiltration is increased in TNBS colitis [32]. This technique can be easily combined with reporter mice and promises to increase the understanding of the mechanisms involved in immune cell migration from blood vessels into the lower GI tract in IBD. 

### 3.2. Microbiota

Recent improvements in high-throughput “omics” technology have also revealed the involvement of the microbiome in IBD. The general consensus indicates that active IBD is characterized by an overall loss of diversity coupled with a dysbiotic phenotype primarily depicted by elevated Proteobacteria and decreased Firmicutes, although inconsistencies between studies remain [33,34,35]. Functionally, the major classes of bacteria affected relate to the production of short-chain fatty acids (e.g., *Faecalibacerium prausnitzii*), bacteria with mucolytic activity (e.g., *Ruminococcous torques*), and sulfate-reducing species including *Desulfovibrio* [36,37,38]. While this dysbiotic composition may contribute to disease progression through dysregulated epithelial barrier function and aberrant immune signaling, it is currently contentious whether dysbiosis itself causes IBD or simply reflects the disease course [39].

Uncovering the compositional shifts in the microbiome has undoubtedly increased our understanding of its role in disease initiation, progression, and relapse. However, techniques such as 16S pyrosequencing are inherently limited by the variations that exist in aspects of their methodology, including inconsistent or incomplete reference libraries, the unknown impact of batched analyses, nonstandardized statistical analyses, and an inability to adequately appreciate the heterogeneity in the microbiome [40]. This is further confounded by the fact that the annotation is based on putative association of the 16S rRNA gene, with a taxon defined as an operational taxonomic unit (OTU) [41,42]. Although whole-genome shotgun sequencing has enhanced our ability to define species taxa with more accuracy, it remains an expensive alternative to 16S and requires significantly greater data analysis [40]. As such, conclusions regarding the functional implications of compositional shifts remain difficult to draw, relying on metagenomic inference using phylogenetic investigation of communities by reconstruction of unobserved states (PICRUSt). Nonetheless, changes in microbiota composition offer the intriguing potential to image microbiota as markers of disease severity in IBD.

## 4. PET and SPECT of Specific Mediators in IBD

PET of radioisotope-labelled antibodies is currently being used in multiple clinical trials in cancer to tailor personalized therapies based on specifically targeted diagnostic tests [43,44]. However, immune-PET of IBD is currently limited to preclinical studies. When deciding which inflammatory mediators to image in IBD, it is firstly important to understand which immune processes are likely to be involved in the model of choice, and secondly to understand what the information will provide regarding the initiating, perpetuating, and resolving phases of colitis. 

### 4.1. Imaging Colitis in Animal Models 

Animal models of colitis have significantly improved our understanding of how inflammation develops and perpetuates, highlighting roles for both the innate and adaptive arms of the immune response. There are currently numerous animal models of IBD, including colitis that develops due to primary genetic defects, adoptive transfer of activated immune cells, or is chemically induced [45]. Importantly, most of these models progress directly from acute through to fulminant colitis and therefore are not useful for investigating pathways involved in the resolution of inflammation and tissue remodeling. Nuclear imaging of colitis has so far relied upon two models of chemically induced colitis: the TNBS model and the dextran sulfate sodium (DSS) model. TNBS is administered by enema and inflammation is therefore restricted to the colon. TNBS is a hapten; it is not directly toxic, but instead binds to autologous proteins and luminal microbiota, turning them immunogenic. The colitis that develops is transmural, involves an IL-12-driven T_H_1/T_H_17-type response, and is therefore considered to model aspects of inflammation associated with CD [46]. DSS differs as it is directly toxic to epithelial cells and, when administered by the oral route in drinking water, results in a colitis that takes longer to develop than TNBS. DSS colitis is considered to model aspects of inflammation associated with UC as the colitis results from direct damage to the epithelial layer and is not as deep as that which occurs with the TNBS model. While animal models are useful for emphasizing the importance of specific mediators and mechanisms, it is important to note that findings from animal models can be difficult to translate to human IBD. This is common with most chronic diseases that have a remitting and relapsing course due to the involvement of multiple and overlapping pathways that are intertwined with disease stage as inflammation progresses and regresses, highlighting the requirement for extensive validation in human subjects. 

#### 4.1.1. PET and SPECT of Immune Mediators in Colitis

Cell-secreted immune mediators are generally classified as chemokines or cytokines, with chemokines mediating cell attraction and cytokines mediating immune responses. Table 1 provides an overview of PET/SPECT studies of immune mediators in human IBD and animal models of colitis. The first study investigating whether radiolabeled antibodies are efficacious for imaging colitis was disappointing, as uptake of ^111^In-labelled IgG was substantially lower than ^111^In-labelled leukocytes, the gold standard at the time, and also the newly developed ^111^In-labelled liposomes in TNBS-colitic rabbits [47]. While imaging of liposomes and leukocytes enabled grading of inflammation, the contrast provided from IgG labelling was not sufficient for this to occur. This result was not surprising given the large abundance of IgG in circulating blood and its relative nonselectivity for colitis. Interleukin (IL)-8, more recently renamed CXCL8, is a chemokine produced by innate immune cells, but also cells in other tissues, including epithelial cells and endothelial cells. IL-8 is primarily involved in attracting neutrophils to inflammatory sites, but also attracts other granulocytes, including mast cells. Colonic inflammation in TNBS-treated rabbits was detected more readily with ^99m^Tc-labelled IL-8 than ^99m^Tc-labelled granulocytes [48]. Furthermore, the severity of inflammation was also able to be graded with ^99m^Tc-IL-8, but not with ^99m^Tc-granulocytes. More recently, IL-8 was labelled with ^99m^Tc for SPECT studies and was found to have higher sensitivity for detecting inflammation than endoscopy in a large clinical study of people with IBD, although specificity was lower than for endoscopy [49]. TNF-α is a cytokine that is secreted by a range of immune cells from both the innate and adaptive arms of the immune response and is produced in large amounts in many autoimmune diseases, including IBD. Neutralizing TNF-α with antibodies, for example infliximab, has proven success in treating IBD [50,51]. In preclinical studies, ^99m^Tc-labelled infliximab was able to discriminate between moderate and severe colonic inflammation in TNBS-treated rats [52]. However, enthusiasm for infliximab as a tracer in preclinical studies was hampered by high background uptake in healthy control rats. This study is of importance as it not only revealed TNF-α mediated inflammation, but it also demonstrated target specificity of a clinically useful drug for IBD. This is also particularly significant given the increased development of biologics, particularly antibody-based therapies, but also the relatively recent expansion of personalized medicine and theragnostic approaches toward treating disease. 

#### 4.1.2. Imaging Immune Cells in Colitis

The DSS colitis model is the most commonly used model to image immune cells in colitis. Table 1 provides an overview of PET/SPECT studies of immune cells in human IBD and animal models of colitis. The integrins α_4_ and β_7_ form a heterodimer (α_4_β_7_) which specifically directs the migration, or homing, of immune cells to the gastrointestinal tract via interactions with its ligand, the addressin MAdDCAM-1 [26]. Targeting this pathway has proved successful in IBD with the recent development of vedolizumab, a monoclonal antibody that blocks α_4_β_7_ [56]. Uptake of ^64^Cu-labelled β_7_ antibodies is increased in DSS-colitic mice relative to healthy controls and also to ^64^Cu-labelled IgG isotype negative control, indicating the potential for β_7_ as a tracer for human IBD [53]. However, high levels of background were also observed in nontarget organs including the small intestine and stomach, prompting the development of antibody fragments. In the same model, uptake of ^64^Cu-labelled β_7_ antibody fragments was superior to both whole ^64^Cu-labelled β_7_ antibodies and ^64^Cu-labelled α_4_β_7_, suggesting that antibody fragments may provide enhanced signaling of inflammation in IBD compared to whole antibodies [54]. The concept of increased sensitivity with small antibodies has recently been validated in cancer biology and in inflammation. ^18^F-labelled antibody fragments directed against the antigen-presenting complex major histocompatibility complex (MHC)II were much more sensitive than standard ^18^F-FDG in detecting xenoplanted tumors, even detecting tumors when growth was neither palpable nor visible [57]. Furthermore, ^18^F-labelled antibody fragments directed against CD11b detected complete Freund’s adjuvant (CFA)-induced inflammation in the mouse paw much earlier than standard ^18^F-FDG [57]. Most recently, ^89^Zr-labelled antibody fragments against the T_HELPER_ marker CD4 were observed to have higher uptake in the colon of DSS mice compared to healthy controls [55]. However, uptake was also increased in the spleen and lymph nodes, highlighting the intrinsic difficulties in imaging immune cells during inflammation, as they generally migrate to and from inflammatory sites and lymph nodes, incorporating blood vessels and lymphatics. 

### 4.2. Imaging the Microbiota in Colitis

As our understanding of microbiota–host interactions grows, it is necessary to move beyond current genomics technology and compositional descriptions. This remains a daunting task when imaging specific microbiota in the gut, as it is limited by the diversity of tissue types that need to be maintained, which is of particular importance when considering the sensitivity of the mucous layer to standard fixation methods and the heterogeneity within gut microenvironments. As such, it is critical that a robust protocol including mucous-preserving sample preparation, image segmentation, and quantitative analysis tools be developed. A framework satisfying these criteria was recently developed by Earle et al. [58], where high-resolution quantification of the spatial organization of the gut microbiome revealed that changes in the proximity of microbes to the epithelium are sufficient to induce increased expression of key inflammatory markers, despite negligible shifts in microbiota composition. Most recently, chemical imaging techniques exploiting a blinking surface enhanced Raman spectroscopy (SERS) have been used to visualize bacteria in vitro with exquisite resolution, and have demonstrated the ability to differentiate between the chemical signatures of different bacteria [59]. MRI, CT, fluorescence/bioluminescence imaging, and PET have also been applied to investigate the activity of bacteria in vivo; however, these typically only provide indirect information about bacterial activity by inference from immune function [60,61]. Recent advances exploiting the bacterial uptake of carbohydrates have proven successful for the selective in vivo imaging of bacteria independently of host factors and secondary pathologies [61,62]. Furthermore, specific bacteria have also been labelled with iron oxide nanoparticles for MRI to track bacteria longitudinally during infection [60]. These developments highlight the potential to image specific bacteria during inflammation, but are yet to be applied to IBD.

## 5. Limitations of Radiolabeled Antibodies

Despite the evidence outlined above supporting the potential use of radiolabeled antibodies or immune mediators for in vivo diagnostic imaging, several issues remain that currently limit enthusiasm for widespread use. The primary limitation is economy-based, as specialized equipment is required for the manufacture and detection of radiolabels. These costs are high, but may be mitigated by improved clinical outcomes, including early detection, and the potential development of personalized medicines to improve clinical responses and reduce side effects. Scientific challenges also exist, particularly with relation to the relatively long half-life of antibodies and the time required for them to move through the circulatory system to the site of inflammation and clear after studies have been performed. These issues relate to the increased risk of cancer due to radiation exposure, particularly as IBD patients already have a higher risk of colon cancer. However, PET is considered safe and the effective dose of whole-body ^18^F-FDG was approximately 7 mSv, much less that the 16 mSv used for a routine abdominal–pelvic CT with contrast [63,64]. Notable efforts are underway to reduce circulation time by using pretargeted methods, as explained in more detail below. The relatively large structure of antibodies may lead to difficulties in penetration of the site of inflammation, although this may be mitigated by altering the structure of the antibody, also explained in more detail below. A limitation also relates to the single mode of PET detection, as only a single mediator can be imaged. This is problematic in terms of detailed studies of immune cell subsets, as they are typically identified by flow cytometry techniques requiring multiple targets. Furthermore, mediators must be accessible to antibodies and are therefore restricted to soluble cytokines and chemokines or markers located on the outer surface of the cell membrane. While intracellular cytoplasmic cytokines and nuclear transcription factors such as FOXP3 (T_REG_) and ROR-γ (T_H_17) are increasingly being used to characterize immune cell subsets, antibody penetration inside immune cells typically requires cell permeabilization, which is not possible in vivo. 

## 6. Future Directions for Imaging IBD

Remarkable progress has been achieved in recent years toward understanding the mechanisms underlying IBD. This has resulted in a number of specific biologics for treatment, but they are yet to be applied to diagnosis or surveillance. The complex immunological mechanisms of IBD indicate that there are multiple markers that could potentially be used for diagnostic imaging and drug bioavailability. Imaging specific sites of inflammation should be achievable relatively easily; however, determining markers that differentiate between IBD and other gastrointestinal diseases, such as cancer, is more problematic and requires extensive preclinical validation. 

Intact antibodies are increasingly being used as therapeutics; however, their utility as imaging agents is reduced by their extended half-life, as the duration of time required to reach a sufficient signal-to-noise ratio is long. Furthermore, extended half-lives may also lead to other harmful biological activity and side effects, potentially altering the biological function being imaged; however, in theory, this risk is minor as concentrations are much typically much lower for imaging than for treatment. Recent developments in antibody engineering include a shift from whole IgG antibodies to fragments, enhancing their physical properties and leading to enhanced efficacy as extensively reviewed in [65]. Enzymatic cleavage or restructuring of the antibody attenuates problems associated with half-life by decreasing clearance times. Furthermore, decreasing the amount of time required to reach a suitable signal also permits the use of short-lived isotopes, reducing radiation exposure.

Pretargeting strategies have also emerged as a means of improving efficacy whilst reducing radiation dose; for example, by using a novel biorthogonal inverse electron demand Diels–Alder reaction between a ^64^Cu-labelled radioligand and a site-specific immunoconjugate [66]. This occurs in four steps: (a) injection of the modified antibody, (b) accumulation of the antibody at its target site and clearance from the blood, (c) injection of the radioligand 24 h later, and (d) the in vivo click ligation between the immunoconjugate and radioligand and subsequent excretion of excess radioligand. The target was able to be delineated four hours post-injection of the radioligand in tumor tissue, with image contrast improved over time and an ideal signal-to-noise ratio at 24 h. This pretargeting approach and similar approaches may expedite the clinical translation of future immunoconjugates for imaging. 

A tantalizing approach is the potential to image different bacterial species; however, this is again currently limited by the lack of knowledge of functional roles for specific microbiota in IBD and the limited ability of PET to simultaneously label multiple targets. Furthermore, imaging of microbiota in general currently requires models where the bacteria can be isolated, labelled, and readministered. Future developments including the ability to use multiple labels, but also advances in the identification of bacterial species of interest and anaerobic culturing of microbiota, would be a welcome addition to preclinical models of IBD in the first instance before clinical studies are warranted. Furthermore, the potential to exploit factors that are selectively produced by bacteria and differentiate between active and quiescent states may provide intriguing insights. Bacterial toxins may be the ideal sensor as they are typically only secreted during disease, are readily identifiable by antibodies, and are relatively easily attached to various detection technologies. However, biosensing technology remains limited in its ability to provide insight into the spatial organization of the microbiome, and as such, the ideal microbial analysis tool remains elusive and current investigations must combine genomic, visual, and activity-based measures.

## 7. Summary

Imaging has a critical role in the diagnosis and management of IBD patients, where endoscopic studies are currently the gold standard. However, there is a clear need for new technologies that are less invasive than endoscopy, but more penetrative at both the local level within the intestinal wall and more broadly along the entire intestinal length. These factors are most relevant for pediatric IBD, where risks of adverse outcomes are increased, and also in small intestinal inflammation and fibrosis, which is generally not accessible by endoscopy. Animal studies indicate that PET and SPECT of immune cells and/or mediators are potentially useful for IBD management and diagnosis. The potential targets for PET/SPECT of immune mediators and cells in active IBD are numerous; however, options for imaging of microbiota are currently limited by the lack of understanding of the role that specific microbiota contribute toward IBD. Translation of these technologies into human studies in IBD is currently nonexistent, yet applicability in other diseases such as cancer is encouraging. Imaging of currently available biologics should be relatively achievable in the short term, while longer-term goals include the development of tracers with deep tissue penetration and short half-lives. Concurrent research into the immune mechanisms underlying IBD should deliver targets that offer insight into disease mechanisms, for example in pediatric IBD, and can differentiate between diseases and/or disease states, for example between active inflammation and fibrosis in UC and CD. In conclusion, the future for the development of imaging techniques in IBD is bright, and further refinement will clearly lead to benefits in the diagnosis and management of IBD patients. 

## Figures and Tables

**Table 1:** Summary of PET/SPECT of human IBD and animal models of colitis. - Not suitable for colitis imaging, + satisfactory image quality, ++ excellent image quality.

Target	Tracers	Species	Model	Outcome	Reference
**Leukocytes**	^99m^Tc-HMPAO-leukocytes	Humans	UC	+	[21]
**CXCL8**	^99m^Tc-CXCL8	Humans	CD and UC	+	[49]
**β_7_**	^64^Cu-FIB504.64-Fab	Mice	DSS	+	[53]
**α_4_β_7_**	^64^Cu-DATK32	Mice	DSS	+	[54]
**β_7_**	^64^Cu-FIB504.64-Fab			+	
**β_7_**	^64^Cu-FIB504.64-F(ab′)_2_ (fragments)			++	
**CD4**	^89^Zr-GK1.5 cys-diabody	Mice	DSS	++	[55]
**TNF-α**	^99m^Tc-Infliximab	Rats	TNBS	+	[52]
**IgG**	^111^In-IgG	Rabbits	TNBS	-	[47]
**Leukocytes**	^111^In-WBC			++	
**Liposomes**	^111^In-liposomes			+	
**IL-8**	^99m^Tc-HYNIC-IL-8	Rabbits	TNBS	++	[48]
**Granulocytes**	^99m^Tc-HMPAO-Granulocytes			+	

Abbreviations: UC: ulcerative colitis. CD: Crohn’s disease. DSS: dextran sulphate sodium. TNBS: tri-nitro benzene sulphonic acid. WBC: white blood cell. Tc: technetium.

## Abbreviations

^64^Cu	Copper-64
^18^F	Fluorine-18
^89^Zr	Zirconium-89
^99m^Tc	Technetium-99m
^111^In	Indium-111
CD	Crohn’s disease
CD4	Cluster of differentiation 4
CD11b	Cluster of differentiation 11b
CFA	Complete Freund’s adjuvant
CT	Computed tomography
CXCL8	C-X-C motif chemokine ligand 8; interleukin 8
DSS	Dextran sodium sulfate
GAP	Goblet cell-associated passage
GI	Gastrointestinal
GIT	Gastrointestinal tract
Fab	Fragment antigen-binding
HMPAO	Hexamethylpropyleneamine oxime
HYNIC	Hydrazinonicotinic acid
IBD	Inflammatory bowel disease
IgG	Immunoglobulin G
IFN	Interferon
IL	Interleukin
JAK	Janus kinase
MAdCAM-1	Mucosal vascular addressin cell adhesion molecule 1
MHCII	Major histocompatibility complex II
MMP	Matrix metalloproteinase
MRI	Magnetic resonance imaging
mSv	Milliseiverts
OTU	Operational taxonomic unit
PET	Positron emission tomography
rRNA	Ribosomal ribonucleic acid
SERS	Surface enhanced Raman spectroscopy
SPECT	Single photon emission computed tomography
TNBS	Trinitro benzene sulfonic acid
TNF-α	Tumor necrosis factor alpha
UC	Ulcerative colitis
VHH	Variable domain of heavy-chain antibodies
WBC	White blood cell
